# Experimental study of brachial plexus and vessel compression: evaluation of combined central and peripheral electrodiagnostic approach

**DOI:** 10.18632/oncotarget.16817

**Published:** 2017-04-04

**Authors:** Chaoqun Yang, Jianguang Xu, Jie Chen, Shulin Li, Yu Cao, Yi Zhu, Lei Xu

**Affiliations:** ^1^ Department of Hand Surgery, Huashan Hospital, Fudan University, Shanghai, China

**Keywords:** brachial plexus, electrodiagnosis, electrophysiology, experimental study

## Abstract

**Introduction:**

We sought to investigate the reliability of a new electrodiagnostic method for identifying Electrodiagnosis of Brachial Plexus & Vessel Compression Syndrome (BPVCS) in rats that involves the application of transcranial electrical stimulation motor evoked potentials (TES-MEPs) combined with peripheral nerve stimulation compound muscle action potentials (PNS-CMAPs).

**Results:**

The latencies of the TES-MEP and PNS-CMAP were initially elongated in the 8-week group. The amplitudes of TES-MEP and PNS-CMAP were initially attenuated in the 16-week group. The isolateral amplitude ratio of the TES-MEP to the PNS-CMAP was apparently decreased, and spontaneous activities emerged at 16 weeks postoperatively.

**Materials and Methods:**

Superior and inferior trunk models of BPVCS were created in 72 male Sprague Dawley (SD) rats that were divided into six experimental groups. The latencies, amplitudes and isolateral amplitude ratios of the TES-MEPs and PNS-CMAPs were recorded at different postoperative intervals.

**Conclusions:**

Electrophysiological and histological examinations of the rats’ compressed brachial plexus nerves were utilized to establish preliminary electrodiagnostic criteria for BPVCS.

## INTRODUCTION

Electrodiagnosis of Brachial Plexus & Vessel Compression Syndrome (BPVCS) is a set of clinical syndromes that are characterized by aching pain, numbness, inertia, and muscle atrophy in the upper extremities due to congenital or postnatal compression of the brachial plexus in the thoracic outlet. Due to the poor prognosis, which includes irreversible intrinsic muscle atrophy in the hands, early diagnosis and intervention are needed to elicit curative effects in patients.

The diagnosis of BPVCS is currently controversial; thus, additional research on the electrodiagnosis of this condition is needed. Urschel [1, 2] suggested a representative electrophysiological diagnostic criterion for TOS based on the detection and analysis of the motor nerve conduction velocities of the ulnar nerve at four different points in the upper extremity. However, other scholars criticized this diagnosis criterion based on the lower accuracy of electrophysiological diagnoses compared with clinical diagnoses [3–7]. Somatosensory evoked potentials (SEPs) are part of the electrodiagnosis and exhibit a high specificity but an unsatisfactory sensitively [8–11]. In previous studies, tests of nerve action potentials (NAPs) in the medial antebrachial cutaneous nerve and other sensory tests are highly sensitive methods for the diagnosis of TOS [12, 13]. Besides, assessments of the functional statuses of compressed motor nerve fibers are also important as assessments of the sensory nerve fibers, and electrophysiological parameters of the motor fibers are the primary data used to detect neurogenic TOS. The combination of MEP and CMAPs is certainly an effective approach to assess TOS, and timulation of proximal segments of peripheral nerves (in humans) is not always straightforward, so MEPs obtained with direct electric stimulation of the scalp can overcome this limitation. Confirmatory tests based on imaging techniques, such as magnetic resonance imaging (MRI), may have poor diagnostic specificity due to the anatomical abnormalities associated with TOS that are frequently observed in asymptomatic individuals [14, 15].

Transcranial electrical stimulation motor evoked potentials (TES-MEPs) have provided insight into motor physiology and pathophysiology and have revealed high incidences of abnormalities in patients with many neuropathies [16–19]. Although the TES-MEP technique is used, it has thus far rarely been applied to the diagnosis of TOS. In the present study, experiments were conducted to diagnose neurogenic TOS in rats based on the combination of TES-MEPs with traditional peripheral nerve stimulation compound muscle action potentials (PNS-CMAPs). Specifically, the goals of this study were the following: 1) to determine the regularity and early specificity of changes in denervation in completely compressed nerve trunks, particularly in terms of the functional transformations of the involved motor nerve fiber, 2) to demonstrate the safety and reliability of the TES-MEP technique in the electrodiagnosis of neurogenic TOS, 3) to further identify an accurate electrophysiological method for detecting the functional statuses of all of the involved brachial plexus nerve roots, and 4) to preliminarily determine the accuracy and reliability of the new electrodiagnosis method for neurogenic TOS in rats based on histological examinations of compressed nerve tissues.

## RESULTS

The alterations in the latencies and amplitudes of the TES-MEPs and PNS-CMAPs of a variety of target muscles at different checkpoints of the compressed brachial plexus nerves are listed in Tables 1–5. The alterations in the regularities of the isolateral amplitude ratios of the TES-MEP to the PNS-CMAPs (Acor:Aper) are illustrated in Table 6.

**Table 1:** Detection of TES-MEP and PNS-CMAP for bilateral infraspinatus muscle in different compressed time course

Time	Stimulation	Latency (ms)		Amplitude (mv)	
(week)	Point	Left	Right	Left	Right
2	Erb	1.46 ± 0.10	1.50 ± 0.11	7.21 ± 0.56	7.20 ± 0.61
	scalp	1.76 ± 0.24	1.80 ± 0.20	7.00 ± 0.67	7.10 ± 0.71
4	Erb	1.56 ± 0.11	1.60 ± 0.10	7.22 ± 0.61	7.00 ± 0.71
	scalp	1.80 ± 0.20	1.86 ± 0.21	7.11 ± 0.54	6.80 ± 0.71
8	Erb	1.54 ± 0.11	1.65 ± 0.13	7.10 ± 0.78	6.84 ± 0.69
	scalp	1.82 ± 0.25	2.30 ± 0.20*	7.00 ± 0.56	6.55 ± 0.80
12	Erb	1.50 ± 0.11	1.70 ± 0.12*	7.15 ± 0.66	6.85 ± 0.74
	scalp	1.80 ± 0.22	2.60 ± 0.23**	7.10 ± 0.56	5.90 ± 0.56
16	Erb	1.52 ± 0.12	1.95 ± 0.12*	7.16 ± 0.60	6.60 ± 0.65
	scalp	1.90 ± 0.21	2.90 ± 0.21**	7.00 ± 0.55	5.10 ± 0.41*
20	Erb	1.52 ± 0.12	2.10 ± 0.10**	7.20 ± 0.78	5.20 ± 0.41*
	scalp	1.85 ± 0.22	3.30 ± 0.15***	7.11 ± 0.54	4.00 ± 0.55**

All data are displayed as mean ± SD. *N* = 6 for 2,4,8,12, and 16 week groups, and *n* = 5 for 20 week group. Erb means superior trunk stimulation position of PNS-CMAP; Scalp means stimulation position of MEP * indicates *P* < 0.05; ** indicates *P* < 0.01; *** indicates *P* < 0.001.

**Table 2:** Detection of TES-MEP and PNS-CMAP for bilateral deltoid muscle in different compressed time course

Time	Stimulation	Latency (ms)		Amplitude (mv)	
(week)	Point	Left	Right	Left	Right
2	Erb	2.46 ± 0.10	2.50 ± 0.11	9.21 ± 0.51	9.20 ± 0.62
	scalp	2.70 ± 0.12	2.71 ± 0.13	8.80 ± 0.61	8.78 ± 0.55
4	Erb	2.50 ± 0.11	2.64 ± 0.12	9.21 ± 0.61	9.10 ± 0.71
	scalp	2.71 ± 0.12	3.00 ± 0.12	8.82 ± 0.56	8.25 ± 0.71
8	Erb	2.52 ± 0.10	3.00 ± 0.12	9.10 ± 0.55	8.90 ± 0.65
	scalp	2.75 ± 0.11	3.43 ± 0.12*	8.89 ± 0.54	7.80 ± 0.56
12	Erb	2.50 ± 0.12	2.82 ± 0.11	9.12 ± 0.54	8.20 ± 0.65
	scalp	2.74 ± 0.11	3.96 ± 0.13**	8.90 ± 0.48	6.80 ± 0.45
16	Erb	2.55 ± 0.10	3.20 ± 0.12*	9.10 ± 0.56	7.42 ± 0.55
	scalp	2.70 ± 0.13	4.00 ± 0.12**	8.85 ± 0.44	5.90 ± 0.52*
20	Erb	2.55 ± 0.11	3.80 ± 0.13**	9.21 ± 0.55	6.30 ± 0.45*
	scalp	2.70 ± 0.10	4.20 ± 0.11***	8.90 ± 0.48	4.50 ± 0.42**

**Table 3:** Detection of TES-MEP and PNS-CMAP for bilateral biceps muscle in different compressed time course

Time	Stimulation	Latency (ms)		Amplitude (mv)	
(week)	Point	Left	Right	Left	Right
2	Erb	2.36 ± 0.10	2.36 ± 0.11	8.11 ± 0.55	8.25 ± 0.51
	scalpl	2.74 ± 0.11	2.73 ± 0.12	7.75 ± 0.62	7.72 ± 0.55
4	Erb	2.40 ± 0.11	2.50 ± 0.11	8.22 ± 0.55	8.00 ± 0.52
	scalp	2.72 ± 0.12	3.00 ± 0.12	7.72 ± 0.66	7.20 ± 0.48
8	Erb	2.44 ± 0.12	2.65 ± 0.11	8.30 ± 0.52	7.80 ± 0.55
	scalp	2.71 ± 0.13	3.42 ± 0.12*	7.70 ± 0.62	6.80 ± 0.52
12	Erb	2.46 ± 0.12	2.86 ± 0.12	8.32 ± 0.55	7.40 ± 0.54
	scalpl	2.73 ± 0.11	3.90 ± 0.13**	7.72 ± 0.60	5.90 ± 0.45
16	Erb	2.45 ± 0.12	3.10 ± 0.11*	8.30 ± 0.52	6.70 ± 0.55
	scalp	2.74 ± 0.12	4.00 ± 0.12**	7.62 ± 0.66	4.75 ± 0.44*
20	Erb	2.44 ± 0.12	3.78 ± 0.11**	8.20 ± 0.51	5.40 ± 0.35*
	scalp	2.72 ± 0.11	4.23 ± 0.12***	7.70 ± 0.55	3.95 ± 0.45**

**Table 4:** Detection of TES-MEP and PNS-CMAP for bilateral abductor pollicis brevis muscle in different compressed time course

Time	Stimulation	Latency (ms)		Amplitude (mv)	
(week)	Point	Left	Right	Left	Right
2	MUAun	3.10 ± 0.10	3.00 ± 0.11	1.51 ± 0.36	1.50 ± 0.31
	scalp	4.40 ± 0.11	4.42 ± 0.12	1.35 ± 0.23	1.35 ± 0.22
4	MUAun	3.11 ± 0.11	3.12 ± 0.12	1.48 ± 0.32	1.48 ± 0.33
	scalp	4.45 ± 0.12	5.60 ± 0.11	1.32 ± 0.25	1.10 ± 0.20
8	MUAun	3.00 ± 0.12	3.40 ± 0.13	1.45 ± 0.32	1.30 ± 0.35
	scalp	4.50 ± 0.11	5.62 ± 0.11*	1.28 ± 0.23	1.05 ± 0.22
12	MUAun	3.10 ± 0.10	3.60 ± 0.12	1.48 ± 0.34	1.20 ± 0.34
	scalp	4.52 ± 0.10	6.90 ± 0.12**	1.28 ± 0.22	0.95 ± 0.21
16	MUAun	3.10 ± 0.12	3.90 ± 0.13*	1.40 ± 0.30	1.15 ± 0.35
	scalp	4.53 ± 0.11	7.00 ± 0.11**	1.30 ± 0.21	0.72 ± 0.25*
20	MUAun	3.34 ± 0.12	4.62 ± 0.12**	1.50 ± 0.30	0.94 ± 0.32*
	scalp	4.50 ± 0.11	7.50 ± 0.11***	1.30 ± 0.23	0.55 ± 0.25**

All data are displayed as mean ± SD.

Abbreviation of MUAmn means stimulation point in the middle upper arm of median nerve.

**Table 5:** Detection of TES-MEP and PNS-CMAP for bilateral abductor digiti minimi muscle in different compressed time course

Time	Stimulation	Latency (ms)		Amplitude (mv)	
(week)	Point	Left	Right	Left	Right
2	MUAun	3.36 ± 0.10	3.40 ± 0.12	1.40 ± 0.26	1.40 ± 0.22
	scalp	4.45 ± 0.12	4.50 ± 0.11	1.20 ± 0.18	1.20 ± 0.19
4	MUAun	3.35 ± 0.11	3.40 ± 0.12	1.45 ± 0.21	1.35 ± 0.20
	scalp	4.50 ± 0.11	5.00 ± 0.11	1.22 ± 0.20	1.10 ± 0.21
8	MUAun	3.36 ± 0.12	3.50 ± 0.11	1.46 ± 0.22	1.25 ± 0.23
	scalp	4.52 ± 0.12	5.56 ± 0.12*	1.18 ± 0.22	1.02 ± 0.22
12	MUAun	3.38 ± 0.11	3.80 ± 0.12	1.47 ± 0.20	1.12 ± 0.22
	scalp	4.55 ± 0.11	6.85 ± 0.12**	1.16 ± 0.23	0.98 ± 0.21
16	MUAun	3.40 ± 0.12	4.20 ± 0.11*	1.50 ± 0.21	1.10 ± 0.25
	scalp	4.50 ± 0.12	7.10 ± 0.11**	1.17 ± 0.22	0.76 ± 0.21*
20	MUAun	3.40 ± 0.11	4.80 ± 0.12**	1.52 ± 0.18	0.91 ± 0.22*
	scalp	4.55 ± 0.12	7.80 ± 0.11***	1.16 ± 0.20	0.55 ± 0.18**

Abbreviation of MUAun means stimulation point in the middle upper arm of ulnar nerve.

**Table 6:** Detection of isolateral amplitude ratio of TES-MEP to PNS-CMAP in different compressed time course (unit: week)

Time course	Superior of BPVCS	Inferior of BPVCS
2	0.98 ± 0.04	0.86 ± 0.04
4	0.96 ± 0.03	0.81 ± 0.03
8	0.93 ± 0.04	0.80 ± 0.04
12	0.88 ± 0.03	0.77 ± 0.04
16	0.62 ± 0.04*	0.65 ± 0.04*
20	0.64 ± 0.03*	0.64 ± 0.05*

**Figure 1:**
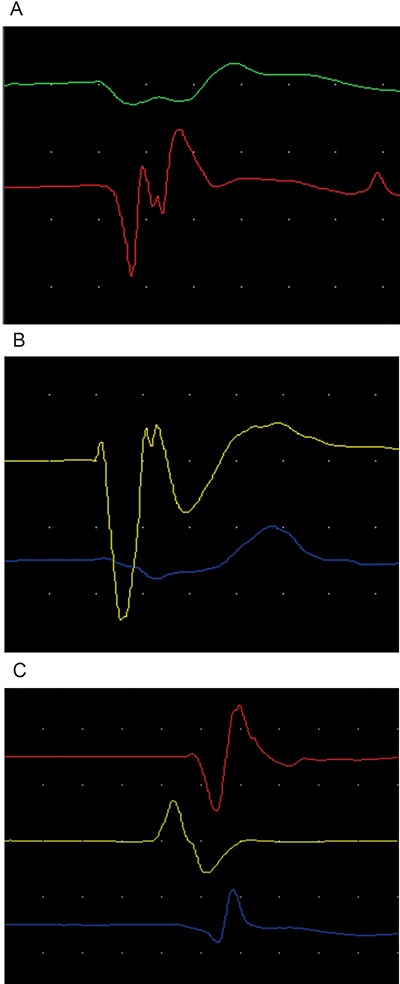
(**A**) represented normal control side electromyography figure of infraspinatus muscle for first line, deltoid muscle for second line and biceps muscle for third line. (**B**, **C**) respectively represented early stage and late stage compressed electromyography figures of infraspinatus muscle, deltoid muscle and biceps muscle dominated by superior trunk of brachial plexus nerve.

**Figure 2:**
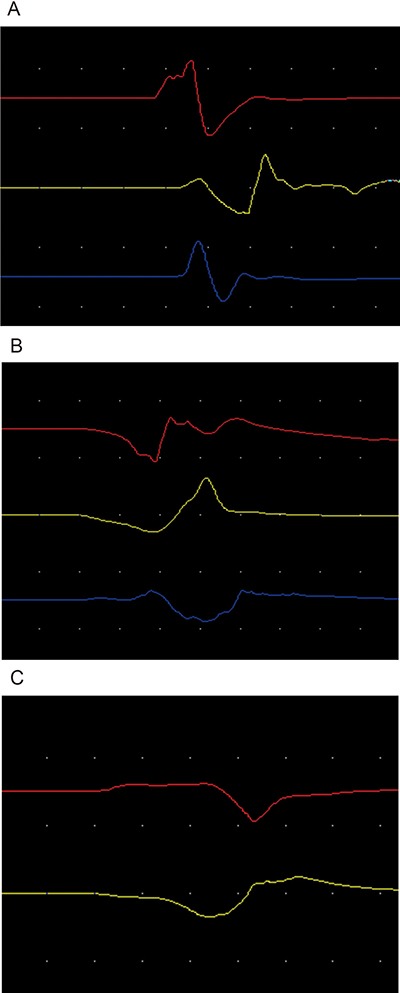
(**A**) represented normal control side electromyography figure of abductor pollicis brevis muscle in the upper line and abductor digiti minimi muscle in the inferior line. (**B**, **C**) respectively represented early stage and late stage compressed electromyography figures of abductor pollicis brevis muscle and abductor digiti minimi muscle dominated by inferior trunk of brachial plexus nerve.

**Figure 3:**
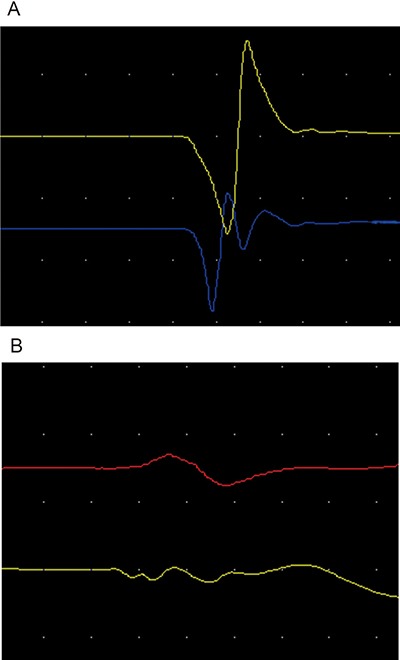
(**A**, **B**) respectively represented early stage and late stage isolateral amplitude ratio of TES-MEP for inferior line to PNS-CMAP for upper line of abductor pollicis brevis muscle dominated by inferior trunk of brachial plexus nerve.

The electrophysiologically detected functional alterations in the motor nerve fibers at the different checkpoint along the brachial plexus nerve can be summarized as follows: 1) The TES-MEP latencies were elongated by 25 ± 1.5% and 45 ± 3.6% compared with the contralateral extremities in the 8- and 12-postoperative-week brachial plexus nerve compression injury groups, respectively (*P* < 0.05 and *P* < 0.01), and the latencies of the PNS-CMAPs were elongated by 10 ± 1.6% and 15 ± 1.8% compared with the contralateral extremities at 8 and 12 weeks, respectively, after brachial plexus nerve compression injuries. However, these differences were not significant (*P* > 0.05). 2) The TES-MEP amplitudes were attenuated by 30 ± 3.6% and 50 ± 3.4% compared with the contralateral extremities at weeks 16 and 20, respectively (*P* < 0.05 and *P* < 0.01). The PNS-CMAP amplitudes were attenuated by 20 ± 3.5% and 30 ± 3.6% at weeks 16 and 20, respectively, compared with the contralateral extremities, and this difference was significant (*P* < 0.05) in the 20-week group (Figures 1 and 2). 3) The isolateral amplitude ratios of the TES-MEPs to the PNS-CMAPs were apparently decreased in the week 16 and 20 groups. Moreover, the isolateral amplitude ratios of the TES-MEPs to the PNS-CMAPs in the superior trunk and inferior trunk models were 0.62 ± 0.04 and 0.65 ± 0.04, respectively (*P* < 0.05; Figure 3). 4) The latencies and amplitudes of the TES-MEP and the PNS-CMAP were initially elongated and subsequently attenuated in the week 16 and 20 groups, respectively. 5) Small quantities of spontaneous activities that included fibrillation potentials and positive sharp waves were discharged in the target muscles of the experimental animals in the week 16 group. The spontaneous discharge activities increased with the progression of the brachial plexus nerve compression process.

## DISCUSSION

### Relationship of the electrophysiological and histological studies of the rats’ compressed brachial plexus nerves

The denervation changes in the motor nerve fibers of the whole-trunk-compressed rat brachial plexus nerves were accurately and objectively detected in detail using the TES-MEP technique in the proximal segment of the compressed nerve trunk. The early neuropathies of the compressed nerves were particularly well detected. Therefore, the latency and amplitude changes in the TES-MEPs were important criteria for the accurate diagnoses of neuropathies in the involved nerve roots. The present study revealed that the TES-MEP latency was significantly elongated in the 8-week group (25 ± 1.5%), and the TES-MEP amplitude of was also significantly attenuated in the 16-week group (30 ± 3.6%). Similarly, the histological alterations of the compressed nerve tissues primarily included demyelination in the 8-week and axonal degeneration in the 16-week group [20]. Consequently, we deduce that pathological alterations soon after nerve compression can theoretically be accurately reflected by changes in TES-MEP latencies and that pathological alterations in the late period nerve compression can be accurately reflected by changes in TES-MEP amplitudes.

Additionally, the isolateral amplitude ratios of the TES-MEP to the PNS-CMAP decreased in the 16-week group by 0.62 ± 0.04 in the superior trunk model and 0.65 ± 0.04 in the inferior trunk model. Moreover, these decreases were closely related to histological alterations due to axonal degeneration following compression injury. Therefore, we also deduced that the isolateral amplitude ratio of the TES-MEP to PNS-CMAP is theoretically an important parameter that reflects pathological changes in the late period after nerve compression.

The PNS-CMAP results only reflected the denervation process of the distal segment of the nerve fiber secondary to the proximally compressed nerve trunk as indicated by the increased latency in the 16-week group and the attenuated amplitude in the 20-week group. These respective alterations were delayed by 8 and 16 weeks relative to the changes in the compressed nerve trunks. In other words, the PNS-CMAP latencies and amplitudes exhibited alterations only after secondary demyelination and axonal degeneration of the distal segment of the compressed nerve trunk. Therefore, we demonstrated that the detection value of PNS-CMAP was related to its ability to reflect functional changes in the motor nerve fiber in the late period of compression injury.

Spontaneous discharge activities emerged in the denervated muscle fibers in week 16 after nerve compression injury, which indicated that the compressed nerve fibers began to undergo Wallerian degeneration [21]. These findings were closely related to the results of the histological investigations of the compressed nerve tissues. Therefore, the value of electromyographical examination was related to determining whether the compressed or involved nerve fibers had begun to undergo Wallerian degeneration, and this determination has diagnostic value.

### Clinical significance of the electrophysiological diagnostic criteria

Mackinnon [22] suggested that the number of myelinated nerve fibers in the nerve trunk is associated with the latency of the evoked potential. Hence, elongated latencies indicate that rapidly conducting myelinated fiber is injured or compressed. In contrast, the amplitude is associated with total number of functionally conducting motor fibers in the nerve trunk. The present experimental study verified this perspective.

All of the TES-MEP and PNS-CMAP parameters were normal in the 2- and 4-week groups. The TES-MEP latency initially exhibited significant elongation in the 8-week group. Additionally, the TES-MEP amplitude decreased, and the isolateral amplitude ratio of the TES-MEP to the PNS-CMAP significantly decreased at week 16 following compression injury. In these circumstances, the TES-MEP technique enabled the objective and accurate detection of the degeneration of the involved motor nerve fiber. More importantly, the value of PNS-CMAP examination resided in its ability to reflect the function of the motor nerve fiber in the late period following compression injury, whereas the EMG examination enabled the identification of Wallerian degeneration in the compressed nerve fibers, which currently has definite diagnostic potential.

### Preliminary electrodiagnostic criteria for TOS

Pathological alterations in compressed nerve tissues in the rat brachial plexus can theoretically be classified as early demyelination changes and late axonal degeneration [23, 24, 27]. By analyzing the correlations between the electrophysiological and histological parameters, we established preliminary early- and late-stage electrodiagnostic criteria for BPVCS based on the following pathological classifications: an increase in the TES-MEP latency of 25% compared with the contralateral extremity in the absence of spontaneous activity on EMG examination composed the early-stage electrodiagnostic criteria; and an increase in the TES-MEP latency of 45% and a decrease in the TES-MEP amplitude of 30% compared with the contralateral extremity combined with a TES-MEP to PNS-CMAP amplitude ratio below 0.6 and the emergence of spontaneous activity on EMG examination composed the late-stage electrodiagnostic criteria. [25].

## MATERIALS AND METHODS

### Establishment and observation of the rat compression model

**Figure 4:**
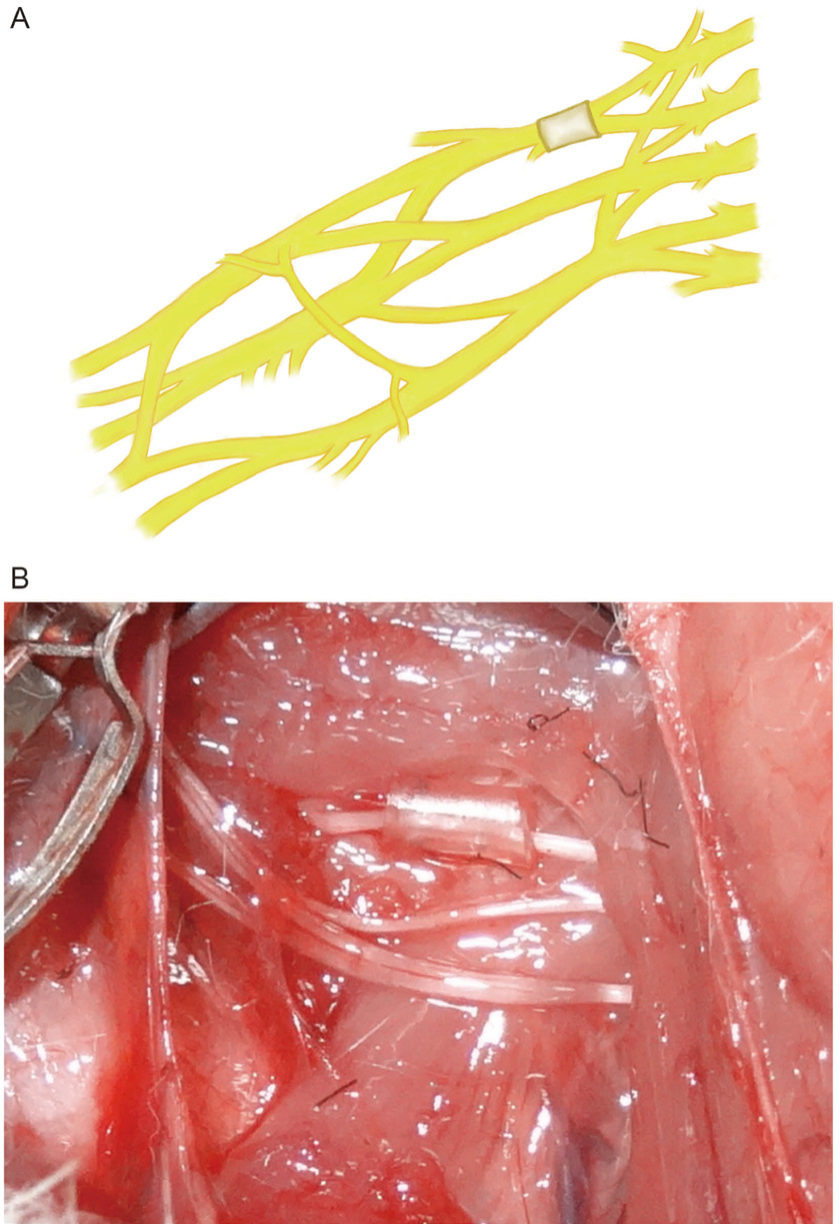
Configuration of superior trunk type model of rat's brachial plexus nerve compression injury

**Figure 5:**
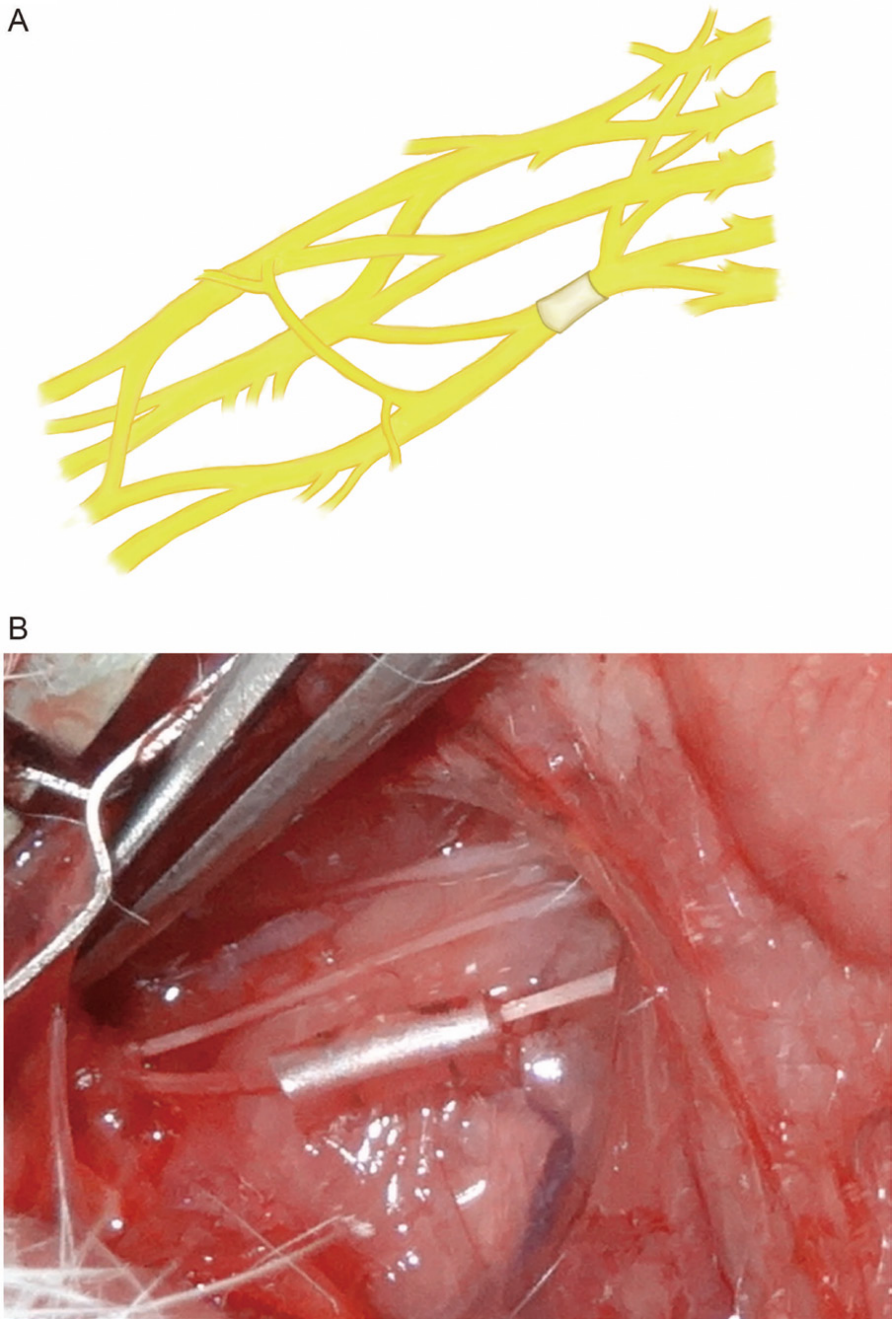
Configuration of inferior trunk type model of rat's brachial plexus nerve compression injury

A total of 72 male SD rats (Slac Laboratory Animal Co., Shanghai, China) were divided into two groups (36/group) as follows: 1) a superior trunk group and 2) an inferior trunk group. The compression model was implemented with medical silica gel tubes (diameter: 1 mm; length: 5 mm). The rats were anesthetized for all surgical procedures via intraperitoneal injections of 1% sodium thiopental, and supplementary doses were administered as needed to maintain a deep state of anesthesia. Transverse anterior cervix incisions were utilized for the surgeries. First, the upper clavicular brachial plexus was exposed, and the external jugular vein, the sternocleidomastoid muscle, the common carotid artery, and the vagal nerve were then surgically dissected away from the median plane. Simultaneously, the anterior cervix muscle and trachea were surgically dissected in the opposite direction. Second, the nerve roots of C5, C6, C7, C8 and T1 were sequentially anatomically exposed and dissected [26]. Finally, the brachial plexus nerve superior trunk chronic compression model was implemented by compressing the right nerve root at C5-6 with a medical silica gel tube (Figure 4), and the brachial plexus nerve inferior trunk chronic compression model was implemented by compressing the right nerve root of C8-T1 (Figure 5). All models were established in the right sides by compressing 10% of the diameter of the nerve trunk, and the left sides were utilized as a normal control group [27].

TES-MEP and PNS-CMAP were utilized to detect the degeneration of the compressed brachial plexus nerves at postoperative weeks 2, 4, 8, 12, 16, and 20. Histological and microstructural studies were performed after the electrophysiological investigations.

### TES-MEP and PNS-CMAP detection conditions and equipment

**Figure 6:**
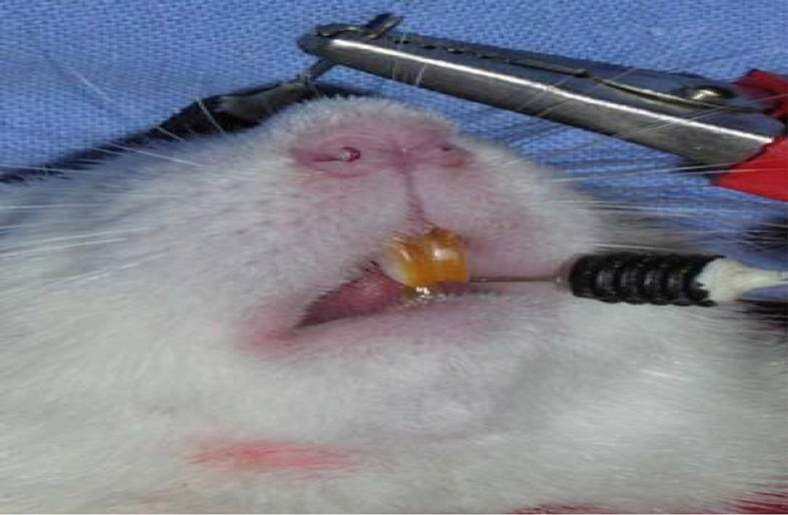
Electrode placing of rat's TES-MEP detection

**Figure 7:**
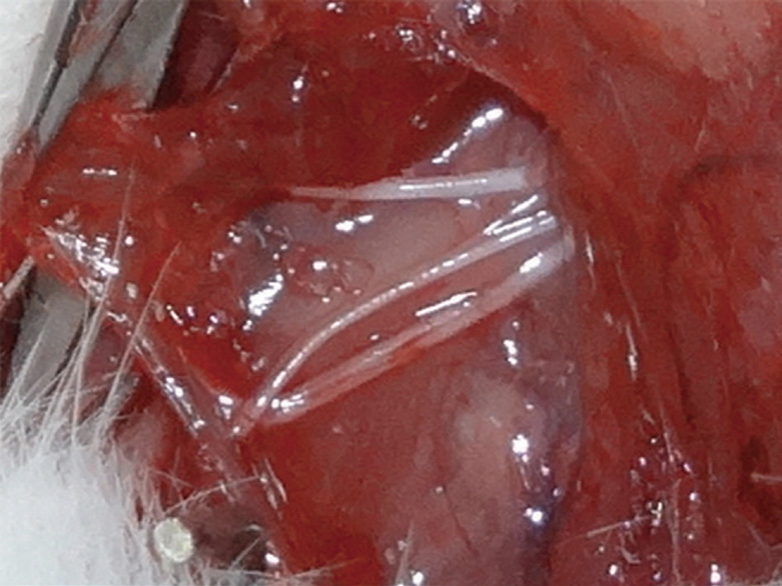
Exposure of median nerve and ulnar nerve in the position of middle upper arm

The examinations of the TES-MEPs combined with PNS-CMAPs were performed with electromyography apparatus as previously reported [1, 11]. Two Argent needles and a concentric needle were used as the stimulating electrodes and recording electrode, respectively. The TES-MEP technique involved placing an anode leaning 0.5 cm over the opposite point of the scalp (Cz) and a cathode over the top of the superior palate in the experimental rat (Figure 6). The TES-MEP stimulation parameters were as follows: frequency, 0.2 ms; sensitivity, 1 mv/D; and current intensity, 10–15 mA. The PNS-CMAP technique involved superior trunk stimulation of the brachial plexus nerve in the superior trunk rat TOS model and stimulation of the medial portion of the upper arm in the inferior trunk rat TOS model (Figure 7). The PNS-CMAP stimulation parameters were as follows: frequency, 0.2 ms; sensitivity, 1 mv/D; and current intensity, 3–5 mA. The recording electrodes were symmetrically and bilaterally placed on the belly of target muscle. The sensitivity of the electromyographical examination was set at 100 μv.

### Recording position and investigational content

For the measurements of the TES-MEPs, PNS-CMAPs, and EMG, the recording electrodes were placed bilaterally on the musculus infraspinatus, deltoid muscle, and biceps muscle in the superior trunk TOS model and on the bilateral abductor pollicis brevis muscle and abductor digiti minimi in the inferior trunk TOS model. The latencies and amplitudes of the TES-MEP and PNS-CMAP action potentials were measured in detail, and the isolateral amplitude ratios of the TES-MEPs to the PNS-CMAPs (Acor:Aper) were simultaneously recorded. The spontaneous activities of the muscle fibers innervated by the compressed nerves were also observed.

### Data analysis

The results are presented as the means ± the SDs. Significant differences were determined one-way ANOVA when 3 groups were compared. Values of *p* < 0.05 were regarded as statistically significant. All statistical analyses were performed using SPASS 11.0 software.

### Ethics

All animal care and experiments were performed with the consent of the Animal Ethics Committee at Shanghai Medical School, Fudan University, and all mice were maintained in a specific pathogen-free environment and fed a standard diet.
